# Supplementary material for: The Breakdown of Stored Triacylglycerols Is Required during Light-Induced Stomatal Opening
Source: Curr Biol. 2016 Mar 7;26(5):707–12. doi: 10.1016/j.cub.2016.01.019 (PMC4791430; doi:10.1016/j.cub.2016.01.019)
Supplement: Document S1. Supplemental Experimental Procedures, Figures S1–S4, and Table S1 [file mmc1.pdf]

Current Biology, Volume *26*

Supplemental Information

The Breakdown of Stored Triacylglycerols Is Required during Light-Induced Stomatal Opening

Deirdre H. McLachlan, Jue Lan, Christoph-Martin Geilfus, Antony N. Dodd, Tony Larson, Alison Baker, Hanna Hõrak, Hannes Kollist, Zhesi He, Ian Graham, Michael V. Mickelbart, and Alistair M. Hetherington

Supplemental Data

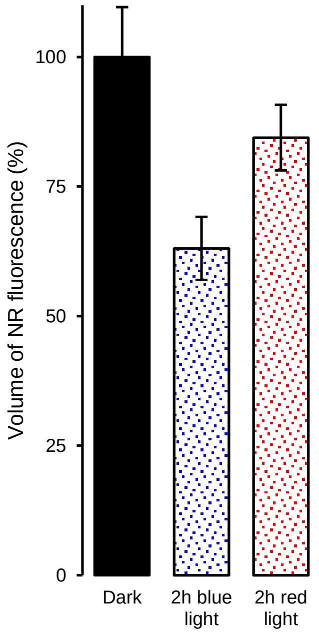

Figure S1. Related to Figure 1. Wavelength specificity of LD reduction. 10 μmol m^{-2} s^{-1} of blue light (λ=470nm) causes significant breakdown of LD ($p<0.01$), while the same amount of red light (λ=660nm) does not ($p>0.05$). Error bars = ± S.E., n=90.

A

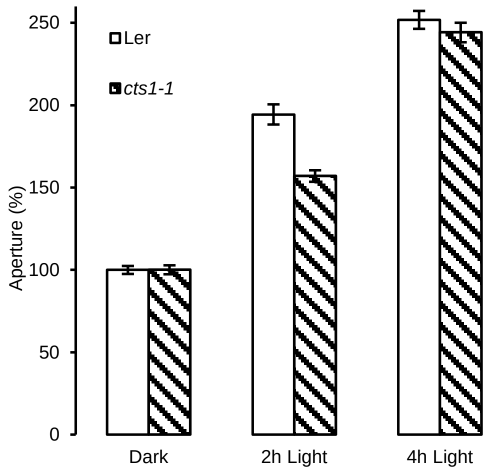

B

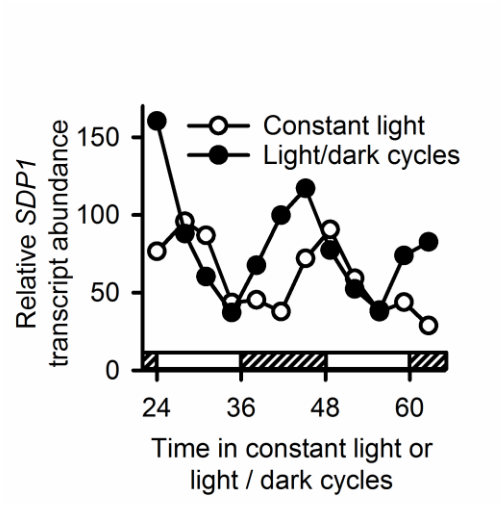

C

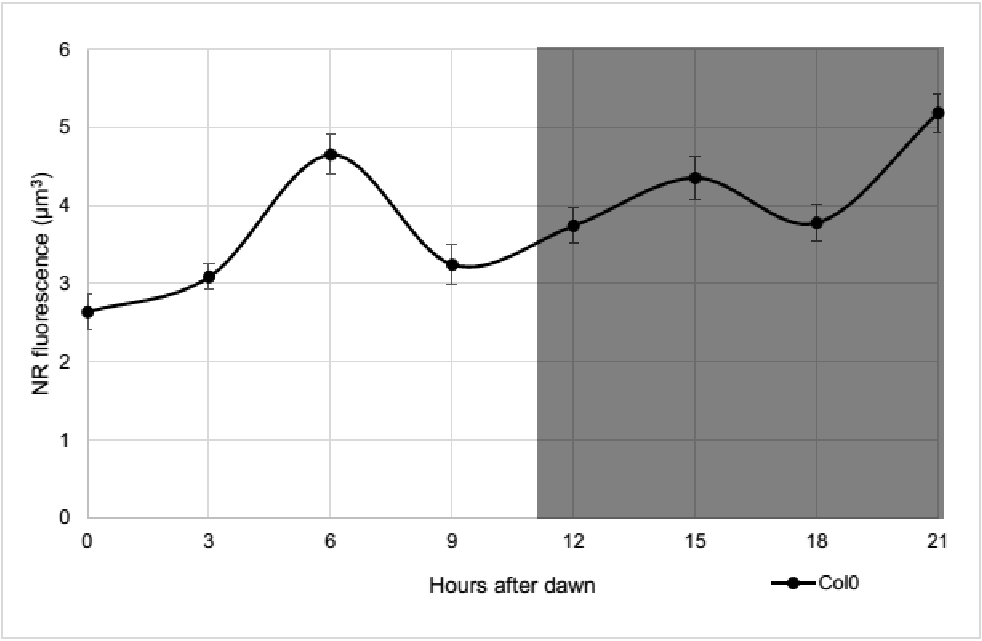

Figure S2. Related to Figure 3. A - The *cts1-1* mutant displays slower light-induced stomatal opening than WT. Error bars = ± SE, n=90, p<0.001 at 2h, p>0.05 at 4h. B - *SDP1* transcript abundance oscillates under light-dark cycles and constant light, peaking around actual or subjective dawn. Data were obtained by interrogation of microarray data using Diurnal [S1]. RNA timecourses were obtained from 35-day old compost grown seedlings [S2] and 8-day old agar grown seedlings [S3] for light/dark and constant light experiments, respectively. C - Change of LD volume in guard cells through a 24 h diurnal cycle. Error bars = ± SE, n=121-142 per timepoint

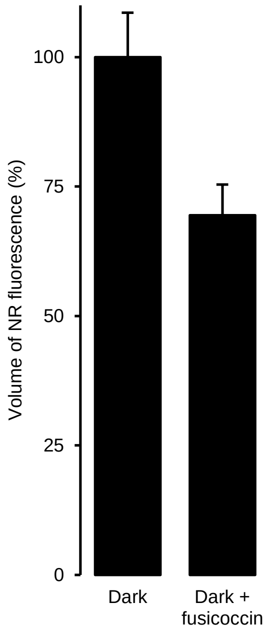

Figure S3. Related to Figure 4. Effect of fusicoccin on LD. Dark-incubated guard cells showed significant LD breakdown after 20μM fusicoccin treatment for 3 h. $p<0.01$, $n=90$, error bars = ± SE.

A

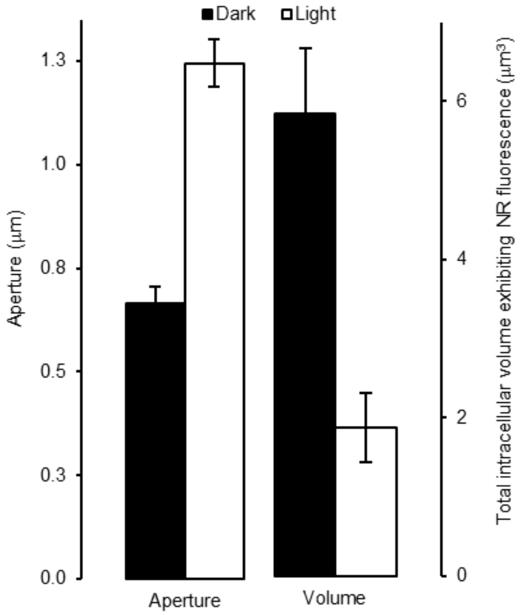

B

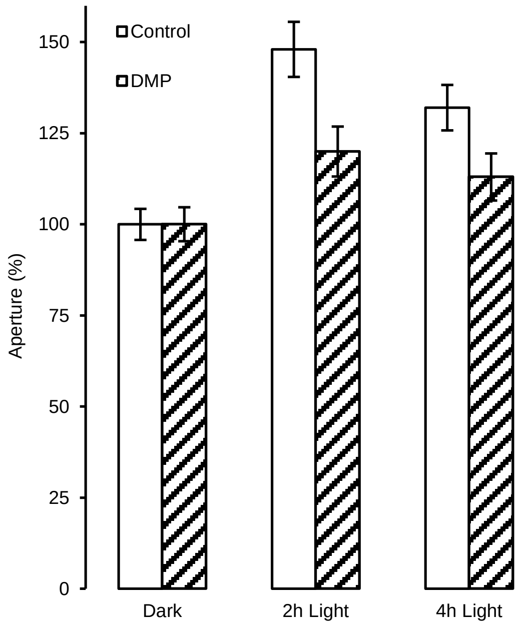

Figure S4. Related to Figure 3. Light-induced stomatal opening in *Selaginella* is associated with a reduction in abundance of LDs and is inhibited by the LD breakdown inhibitor DMP. (A) Light induced stomatal opening is associated with a decrease in NR fluorescence (n=90, $p<0.001$, error bars = ±SE). (B) Light-induced stomatal opening is inhibited by DMP (n=90, $p<0.001$, error bars = ± SE)

Table S1. Abundance of total TAG and individual TAG molecular species pre and post dawn (for full details see methods).

TAG species	Time	ng/mg FW	S.E.	Change (% of predawn)	p value	No. of samples
18:2-18:3-18:3	predawn	3.7	0.65	48	0.011	13
	postdawn	1.8	0.32			14
18:2-18:2-18:3	predawn	2.5	0.41	50	0.013	13
	postdawn	1.3	0.22			14
18:3-18:3-18:3	predawn	1.6	0.31	54	0.048	13
	postdawn	0.9	0.17			14
16:0-18:2-18:2	predawn	1.4	0.08	66	0.032	2
	postdawn	0.9	0.04			2
16:0-18:3-18:3	predawn	0.8	0.19	69	0.267	13
	postdawn	0.6	0.13			14
18:2-18:2-18:2	predawn	1.4	0.17	82	0.314	6
	postdawn	1.2	0.17			8
18:1-18:1-18:2	predawn	2.0	0.94	87	0.786	13
	postdawn	1.7	0.26			14
16:0-16:0-18:2	predawn	0.5	0.16	106	0.883	6
	postdawn	0.5	0.13			8
18:0-18:0-18:2	predawn	0.9	0.13	114	0.498	6
	postdawn	1.1	0.13			8
18:0-18:2-18:3	predawn	0.2	0.05	114	0.645	6
	postdawn	0.3	0.05			8
18:1-18:2-18:2	predawn	1.9	0.42	115	0.644	6
	postdawn	2.1	0.40			8
16:0-18:1-18:2	predawn	1.5	0.43	118	0.644	6
	postdawn	1.8	0.37			8
18:0-18:2-18:2	predawn	0.5	0.15	125	0.531	6
	postdawn	0.6	0.13			8
16:0-18:1-18:1	predawn	2.0	0.65	130	0.478	6
	postdawn	2.6	0.53			8
**Total**	**predawn**	**14.5**	**2.84**	**76**	**0.355**	**13**
	**postdawn**	**11.1**	**2.18**			**14**

Table S2.

Expression levels of TAG lipolysis, peroxisomal fatty acid beta-oxidation, glyoxylate cycle and gluconeogenesis related genes in Col-0 derived guard cell and leaf samples. The genes are grouped according to function and the order proceeds from lipolysis through the various steps and pathways to gluconeogenesis. Raw microarray data was downloaded and independently processed. Fold change was calculated to represent the comparison of the 2 sample types (guard cell and leaf) that had been maintained either in the presence or absence of exogenous ABA. Gene descriptions are from the latest version of the Arabidopsis genome annotation TAIR10 (http://www.arabidopsis.org/portals/genAnnotation/gene_structural_annotation/annotation_data.jsp).

Supplemental Experimental Procedures

Microarray data in Table S2. Unprocessed Affymetrix microarray data from Col-0 guard cell and leaf samples [S4] was downloaded from the gene expression omnibus (GEO) at NCBI using accession No. GSE19520 (http://www.ncbi.nlm.nih.gov/geo/query/acc.cgi?acc=GSE19520). The data were processed and normalised using the MAS5 algorithm in R/bioconductor with a mean target intensity of 500. A comprehensive list of genes involved in TAG lipolysis, peroxisomal fatty acid beta-oxidation, the glyoxylate cycle and gluconeogenesis was assembled from the Arabidopsis Acyl-Lipid Metabolism Website (ARALIP, http://aralip.plantbiology.msu.edu/pathways/triacylglycerol_fatty_acid_degradation,) [S5] and the compiled list of genes involved in storage oil mobilisation [S6]. The CGI-58 gene with a proposed role in neutral lipid homeostasis is also included [S7]. The expression values of these genes were calculated by averaging the triplicate measurements and a fold change was used to represent the comparison of the 2 sample types (guard cell and leaf) that had been maintained either in the presence or absence of exogenous ABA.

Supplemental References

S1. Mockler, T.C., Michael, T.P., Priest, H.D., Shen, R., Sullivan, C.M., Givan, S.A., McEntree, C., Kay, S.A. and Chory, J. (2007). The DIURNAL project: diurnal and circadian expression profiling, model-based pattern matching and promoter analysis. *Cold Spring Harb. Symp. Quant. Biol. 72*, 353-363.

S2. Edwards, K.D., Anderson, P.E., Hall, A., Salathia, N.S., Locke, J.C.W., Lynn, J.R., Straume, M., Smith, J.Q., and Millar, A.J. (2006). FLOWERING LOCUS C mediates natural variation in the high-temperature response of the Arabidopsis circadian clock. Plant Cell *18*, 639-650.

S3. Blasing, O.E., Gibon, Y., Gunther, M., Hohne, M., Morcuende, R., Osuna, D., Thimm, O., Usadel, B., Scheible, W.R., and Stitt, M. (2005). Sugars and circadian regulation make major contributions to the global regulation of diurnal gene expression in Arabidopsis. Plant Cell *17*, 3257-3281.

S4. Wang, R.S., Pandey, S., Li, S., Gookin, T.E., Zhao, Z.X., Albert, R., and Assmann, S.M. (2011). Common and unique elements of the ABA-regulated transcriptome of Arabidopsis guard cells. BMC Genomics *12*:216, doi: 10.1186/1471-2164-12-216.

S5. Li-Beisson Y., Shorrosh B., Beisson F., Andersson M.X., Arondel V., Bates P.D., Baud S., Bird D., DeBono A., Durrett T.P., Franke R.B., Graham I.A., Katayama K., Kelly A.A., Larson T., Markham J.E., Miquel M., Molina I., Nishida I., Rowland O., Samuels L., Schmid K.M., Wada H., Welti R., Xu C. Zallot R., and Ohlrogge J. (2010) Acyl-Lipid Metabolism. *The Arabidopsis Book* 8:e0133. doi:10.1199/tab.0133

S6. Graham, I.A. (2008). Seed storage oil mobilization. Annu Rev Plant Biol *59*, 115-142.

S7. James, C.N., Horn, P.J., Case, C.R., Gidda, S.K., Zhang, D.Y., Mullen, R.T., Dyer, J.M., Anderson, R.G.W., and Chapman, K.D. (2010). Disruption of the Arabidopsis CGI-58 homologue produces Chanarin-Dorfman-like lipid droplet accumulation in plants. P Natl Acad Sci USA *107*, 17833-17838.
