# Supplementary material for: The Breakdown of Stored Triacylglycerols Is Required during Light-Induced Stomatal Opening
Source: Curr Biol. 2016 Mar 7;26(5):707–12. doi: 10.1016/j.cub.2016.01.019 (PMC4791430; doi:10.1016/j.cub.2016.01.019)
Supplement: Document S2. Article plus Supplemental Information [file mmc3.pdf]

Report

Current Biology

The Breakdown of Stored Triacylglycerols Is Required during Light-Induced Stomatal Opening

Graphical Abstract

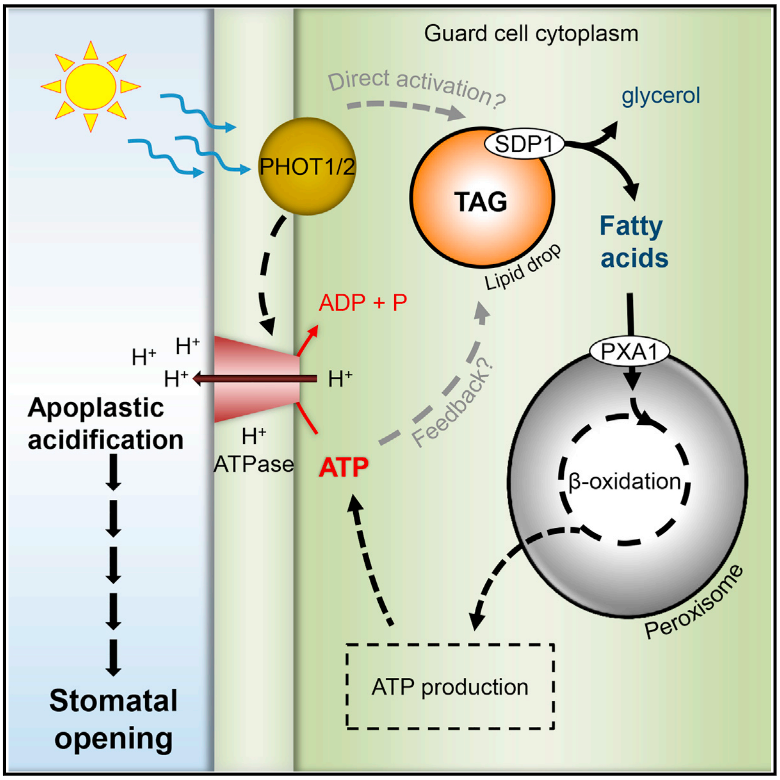

Highlights

- Guard cells break down triacylglycerols to supply ATP for use in stomatal opening
- Light-induced stomatal opening is delayed in triacylglycerol catabolism mutants
- PHOT blue light receptors are involved in reductions in lipid droplet (LD) abundance
- Light-induced reductions in LD abundance occur in *Selaginella* guard cells

Authors

Deirdre H. McLachlan, Jue Lan, Christoph-Martin Geilfus, ..., Ian Graham, Michael V. Mickelbart, Alistair M. Hetherington

Correspondence

alistair.hetherington@bristol.ac.uk

In Brief

Stomata are pores on the surfaces of leaves that regulate gas exchange. McLachlan et al. show that triacylglycerols stored in guard cell lipid droplets are broken down to provide ATP required during light-induced stomatal opening. Experiments with the lycophyte *Selaginella* suggest that, in evolutionary terms, this is a conserved mechanism.

CrossMark

McLachlan et al., 2016, Current Biology *26*, 707–712
March 7, 2016 ©2016 The Authors
http://dx.doi.org/10.1016/j.cub.2016.01.019

CellPress

Current Biology
Report

The Breakdown of Stored Triacylglycerols Is Required during Light-Induced Stomatal Opening

Deirdre H. McLachlan,[1,7,8] Jue Lan,[1,7] Christoph-Martin Geilfus,[2] Antony N. Dodd,[1] Tony Larson,[3] Alison Baker,[4] Hanna Hõrak,[5] Hannes Kollist,[5] Zhesi He,[3] Ian Graham,[3] Michael V. Mickelbart,[1,6] and Alistair M. Hetherington[1,*]
[1]School of Biological Sciences, Life Sciences Building, University of Bristol, 24 Tyndall Avenue, Bristol BS8 1TQ, UK
[2]Institut fur Pflanzenernährung und Bodenkunde, Christian-Albrechts-Universität zu Kiel, Hermann-Rodewald- Straße 2, 24118 Kiel, Germany
[3]Centre for Novel Agricultural Products, Department of Biology, University of York, York YO10 5DD, UK
[4]Centre for Plant Sciences, School of Molecular and Cellular Biology, University of Leeds, Leeds LS2 9JT, UK
[5]Institute of Technology, University of Tartu, Nooruse 1, Tartu 50411, Estonia
[6]Department of Botany and Plant Pathology, Purdue University, 915 W. State Street, West Lafayette, IN 47907, USA
[7]Co-first author
[8]Present address: The Sainsbury Laboratory, Norwich Research Park, Norwich NR4 7UH, UK
*Correspondence: alistair.hetherington@bristol.ac.uk
http://dx.doi.org/10.1016/j.cub.2016.01.019
This is an open access article under the CC BY license (http://creativecommons.org/licenses/by/4.0/).

SUMMARY

Stomata regulate the uptake of CO_2 and the loss of water vapor [1] and contribute to the control of water-use efficiency [2] in plants. Although the guard-cell-signaling pathway coupling blue light perception to ion channel activity is relatively well understood [3], we know less about the sources of ATP required to drive K^+ uptake [3–6]. Here, we show that triacylglycerols (TAGs), present in *Arabidopsis* guard cells as lipid droplets (LDs), are involved in light-induced stomatal opening. Illumination induces reductions in LD abundance, and this involves the PHOT1 and PHOT2 blue light receptors [3]. Light also induces decreases in specific TAG molecular species. We hypothesized that TAG-derived fatty acids are metabolized by peroxisomal β-oxidation to produce ATP required for stomatal opening. In silico analysis revealed that guard cells express all the genes required for β-oxidation, and we showed that light-induced stomatal opening is delayed in three TAG catabolism mutants (*sdp1*, *pxa1*, and *cgi-58*) and in stomata treated with a TAG breakdown inhibitor. We reasoned that, if ATP supply was delaying light-induced stomatal opening, then the activity of the plasma membrane H^+-ATPase should be reduced at this time. Monitoring changes in apoplastic pH in the mutants showed that this was the case. Together, our results reveal a new role for TAGs in vegetative tissue and show that PHOT1 and PHOT2 are involved in reductions in LD abundance. Reductions in LD abundance in guard cells of the lycophyte *Selaginella* suggest that TAG breakdown may represent an evolutionarily conserved mechanism in light-induced stomatal opening.

RESULTS

As lipid droplets (LDs) are found in the guard cells of higher and lower plants [7–9], we decided to investigate whether the oxidation of stored TAGs provides a source of ATP for driving light-induced stomatal opening. First, we used the LD stain Nile Red (NR) [10] to show that *Arabidopsis thaliana* guard cells possess NR-staining material consistent with LDs (Figure 1A). Next, we showed that LD volume decreased significantly ($p < 0.001$) during light-induced stomatal opening (Figure 1B). To investigate whether this response was mediated, at least in part, by the blue light phototropin-signaling pathway, we used the *phot1 phot2* double mutant that is compromised in blue-light-induced stomatal opening [11]. Figure 1C shows that this is indeed the case because both blue-light-induced reduction in LD volume and stomatal opening are decreased significantly ($p < 0.05$) in this background compared with the WT. We confirmed this finding by investigating the effects of blue or red light on LD volume. Figure S1 shows that, compared with darkness, blue light significantly reduced LD volume, whereas the same was not true for red light.

We next investigated the fate of the guard cell triacylglycerol (TAG) fraction during exposure to light. To provide a physiological context for this experiment, we investigated the process of light-induced stomatal opening that occurs at dawn. We collected guard-cell-enriched material at 1 hr pre-dawn and 3 hr post-dawn. During the transition from dark to light, there were significant ($p < 0.05$) reductions in 4 of the 14 detectable TAG molecular species (Figure 2; Table S1). This included 18:2-18:3-18:3 and 18:2-18:2-18:3, which were the most abundant and second most abundant of all the TAG molecular species. Together, the four species that declined accounted for 63% of the TAG species identified in the guard-cell-enriched fraction (pre-dawn).

In plants, TAG breakdown is comparatively well understood through research on oil seeds [12]. Recently, the possible role(s) of TAGs and LDs in vegetative tissues has been attracting considerable interest [13, 14]. In *Arabidopsis* seeds, TAGs are first released from LDs and broken down to their constituent fatty

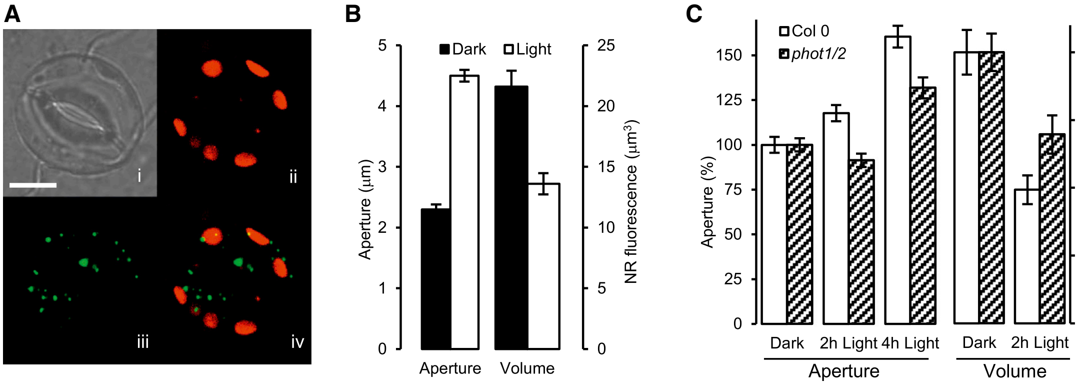

Figure 1. Stomatal Opening Is Associated with a Reduction in Abundance of LDs, and This Response Involves the Blue Light Receptors PHOT1 and PHOT2

(A) Guard cells contain cytoplasmic NR-staining LDs (i, bright field; ii, autofluorescence; iii, NR fluorescence; iv, overlay of ii and iii; scale bar, 5 μm).
(B) Light-induced stomatal opening is associated with a decrease in NR fluorescence (n = 120 for each; $p < 0.001$ at 4 hr for both; error bars represent ±SE).
(C) Light-induced stomatal opening is disrupted in the *phot1/phot2* double mutant, as is LD breakdown as estimated by NR fluorescence (n = 90 for aperture; n = 75–95 for volume; $p < 0.001$ at 2 hr and 4 hr for stomatal opening and $p < 0.05$ for LD reduction).
See also Figure S1.

acids and glycerol by the TAG lipase SUGAR DEPENDENT1 (SDP1) [15]. The fatty acids are subsequently imported into the peroxisome by the ABC transporter COMATOSE (CTS)/ PEROXISOMAL ABC TRANSPORTER 1 (PXA1) [16–18], after which they enter the β-oxidation cycle. Indeed, it has previously been suggested that, in leaves, chloroplasts are a source of fatty acids that are metabolized in peroxisomes and contribute to ATP production [19]. Although it is well established that guard cells contain peroxisomes [20], much less is known about the capacity of these cells for TAG catabolism. Using an in silico approach, we found that genes encoding enzymes involved in TAG breakdown and fatty acid β-oxidation are expressed in guard cells (Table S2). If our hypothesis that TAG breakdown is required for stomatal opening is correct, we reasoned that plants with lesions in TAG breakdown and metabolism should have aberrant light-induced stomatal opening. To investigate this, we studied this process in isolated epidermal strips of well-characterized independent mutant alleles of *SDP1* (*sdp1-4* and *sdp1-5*), *PXA1* (*pxa1* and *cts-1*) [15–18], and the PXA1 regulator *COMPARATIVE GENE IDENTIFICATION-58* (*cgi-58*) [21, 22]. The data in Figure 3A show that, in *sdp1-4* and *sdp1-5*, stomatal opening lagged significantly ($p < 0.05$ for both) behind WT plants after 2 hr incubation in the light, whereas after 4 hr incubation, there was no significant difference between the mutants and WT. Significantly ($p < 0.001$) reduced stomatal opening also occurred in the *cgi-58*, *pxa1*, and *cts-1* mutants (Figures 3B, 3C, and S2). We also investigated LD volume in *pxa1* and *cgi58* 2 hr after a transition from dark to light (Figures 3B and 3C). In *pxa1*, we found no difference from the WT, as would be expected, as this mutant has a lesion in fatty acid uptake into the peroxisome rather than TAG breakdown. In the case of *cgi58*, there was a slightly greater reduction in LD volume compared with the WT ($p < 0.05$).

We sought independent confirmation of these results using different experimental approaches. When we treated stomata in isolated epidermes with diphenyl methylphosphonate (DMP), an inhibitor of LD mobilization that acts early in the β-oxidation pathway [23], we saw significantly ($p < 0.001$) delayed light-induced stomatal opening (Figure 3D). Next, we measured the stomatal conductance of WT and *sdp1-4* and *sdp1-5* plants. For the majority of the light-dark cycle, stomatal conductance between the mutants and WT was not different (data not shown). Strikingly, after 16 min of exposure to light, the WT plants had significantly greater stomatal conductance than either of the *sdp1* alleles (Figure 3E; $p < 0.05$ for both). Interestingly, interrogation of transcriptome data revealed that *SDP1* transcript abundance had a clear peak immediately pre-dawn in daily cycles of light and dark and also constant light (Figure S2). Although this analysis was performed using RNA derived from mature rosette leaves, if the same holds true for guard cells, it suggests there is a role for SDP1 in TAG breakdown during stomatal opening at dawn and that there may be circadian regulation of *SDP1*. In this context, it is interesting to note that guard cell LD volume is at its lowest at around dawn and reaches two maxima; the first is approximately 3 hr before dawn, whereas the second is during the day, some 6 hr after dawn (Figure S2).

One way to test whether TAGs in LDs are metabolized to ultimately produce the ATP required in stomatal opening is to investigate whether the activity of an ATP-consuming enzyme, known to be involved in stomatal opening, is reduced in a TAG metabolism mutant. We focused on the plasma membrane H^+-ATPase, which is activated during blue-light-induced stomatal opening and induces apoplastic acidification in *Arabidopsis* [24]. As a measure of the activity of this enzyme, we used Oregon Green 488-dextran ratio microscopy [25–27] to monitor blue-light-induced apoplastic acidification in *sdp1-4*, *sdp1-5*, *pxa1*, and *cgi58* mutants. The data in Figure 4A show that there is a delay of over 1 hr until the apoplastic pH (pH_{apo}) in the two *sdp1* alleles reached the same value as the WT. pH_{apo} of *pxa1* and *cgi-58* never drops as low as the WT. This response is consistent with the delay in light-induced stomatal opening seen in these mutants (Figures 3B and 3C) and is also consistent with our hypothesis that ATP availability limits both

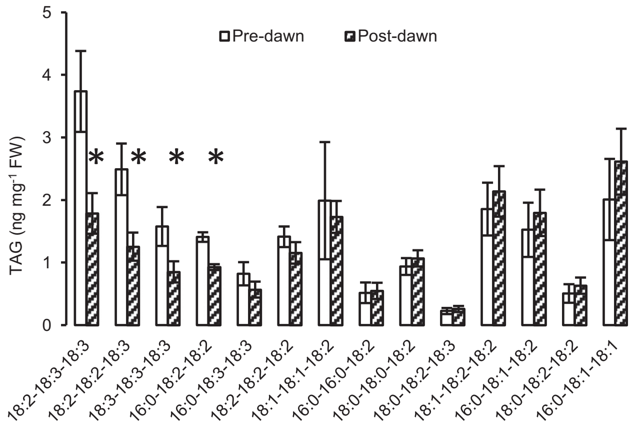

Figure 2. Changes in Abundance of Specific TAG Molecular Species during the Pre- to Post-dawn Transition

Error bars represent ±SE; n = 13–14; significant ($p < 0.05$) changes are indicated by an asterisk.

responses over this time period. We confirmed that there are no differences in the expression of the plasma membrane H^+-ATPase, AHA1 (At2g18960) between *pxa1* and WT using microarray data (data not shown).

Our hypothesis is that TAGs are catabolized and the fatty acids from these TAGs are oxidized to produce ATP required for stomatal opening. Another possibility is that, in addition to providing ATP, TAG breakdown is also used to provide carbon skeletons for malate^{2-} synthesis that also occurs during stomatal opening [3, 4]. In this context, the results from our in silico analysis are revealing (Table S2). The lack of expression of glyoxylate cycle genes in guard cells would suggest that acetyl CoA, the product of β-oxidation, is converted to citrate and used in mitochondrial respiration [28] rather than in gluconeogenesis. As we see delayed light-induced stomatal opening in the *sdp1* mutant, it is unlikely that disruptions to the synthesis of oxylipins such as jasmonate or indole butyric acid, known to be synthesized through the peroxisomal β-oxidation pathway [29–31], underlie the opening phenotype reported here. Although we did not investigate it, an additional possible role for the acyl groups released by lipase action would be as a ready reserve to support the extensive plasmalemma and tonoplast remodeling that occurs during stomatal opening.

We reasoned that, if our hypothesis concerning the role of guard cell TAG catabolism in generating ATP to support stomatal opening was correct, then we should see a greater decline in LD abundance in a genotype that is depleted in an alternative metabolic source of energy for use in stomatal opening. To test this hypothesis, we focused on the *Arabidopsis* starch-deficient phosphoglucomutase (*pgm1*) mutant, which is known to exhibit reduced blue-light-induced stomatal opening [32, 33]. We observed that there was a significantly ($p < 0.001$) greater reduction in LD volume in the *pgm1* mutant when compared with the WT after 2 hr exposure to white light, and light-induced stomatal opening was significantly delayed ($p < 0.05$) in this mutant (Figure 4B). These results are consistent with greater TAG breakdown acting to compensate for the lack of starch in this mutant and, together with the delay in blue-light-induced reductions in pH_{apo}, would seem to favor our metabolic explanation for the effects we observe. Finally, we investigated whether activating the plasmalemma H^+-ATPase directly, in the absence of light, using the fungal toxin fusiccocin (FC), which brings about stomatal opening through activation of the H^+-ATPase [34], would result in a reduction in LD volume. The results presented in Figure S3 show that, in the dark, FC induces a significant reduction in LD volume. This suggests the existence of a mechanism for sensing cytosolic ATP levels. When ATP levels fall below those required to support the requirements of H^+-ATPase activity during stomatal opening, there is a feedback mechanism that results in increased TAG catabolism.

DISCUSSION

The data in this paper highlight the importance of integrating energetic and metabolic components into models of stomatal function. In this context, one of the most pressing questions to arise from our work is how the phototropin-mediated guard-cell-signaling pathway integrates with TAG metabolism and specifically regulates the activity of the SDP1 TAG lipase. Although the majority of our work was carried out in *Arabidopsis* guard cells, we also investigated LD dynamics during light-induced stomatal opening in the lycophyte *Selaginella*. Figures S4A and S4B show that, as in *Arabidopsis*, there is a reduction in LD volume during stomatal opening and that the β-oxidation inhibitor DMP interfered with this process. These data support the suggestion that stomatal opening is an active, energy-requiring process in basal plant lineages [35, 36]. In addition, as the guard cells of other higher plants such as the angiosperms *Campanula*, Pea, the gymnosperm *Abies*, the horsetail *Equisetum* [9], and the moss *Funaria* [37] contain LDs, it is plausible that TAGs have a universal role in guard cell movement in both angiosperms and more basal plant lineages. Previous work has pointed to a role for leaf LDs in the production of antifungal oxylipins [38]. Here, we provide evidence for a new and unexpected role for these structures in guard cells and in doing so identify TAGs as a source for ATP production during stomatal opening.

EXPERIMENTAL PROCEDURES

Plants

Arabidopsis plants were grown under short days (10 hr light:14 hr), 110–120 μmol m^{-2} s^{-1} photon flux density (PFD), and 70% humidity. Experiments were performed on 28- to 35-day-old plants. All lines were germinated on half-strength Murashige and Skoog basal salts media plates with 1% sucrose. *cts-1* had their testa manually ruptured post-stratification. Plants for gas exchange experiments were grown at a 23°C:18°C day:night temperature regime; all other plants were grown at 22°C:20°C. *Selaginella uncinata* was grown under the same conditions with the exception that light was 50 μmol m^{-2} s^{-1} PFD. For the ratiometric pH quantification experiments, *Arabidopsis* was grown as above but at a PFD of 140 μmol m^{-2} s^{-1}.

Stomatal Aperture Measurements

All epidermal strip experiments were performed in 50 mM KCl, 10 mM MES (pH 6.15) at 22°C, and 100 μmol m^{-2} s^{-1} PFD, where applicable. Epidermal strips, detached pre-dawn, were incubated in darkness for 2 hr before transfer to light for 2–4 hr. Strips were then mounted on glass slides, and aperture width was determined on a Leica DM IRB microscope. DMP (TCI Europe NV), when used, was applied 1 hr before the dark to light transition at a concentration of 25 μM (from a 25 mM stock in DMSO).

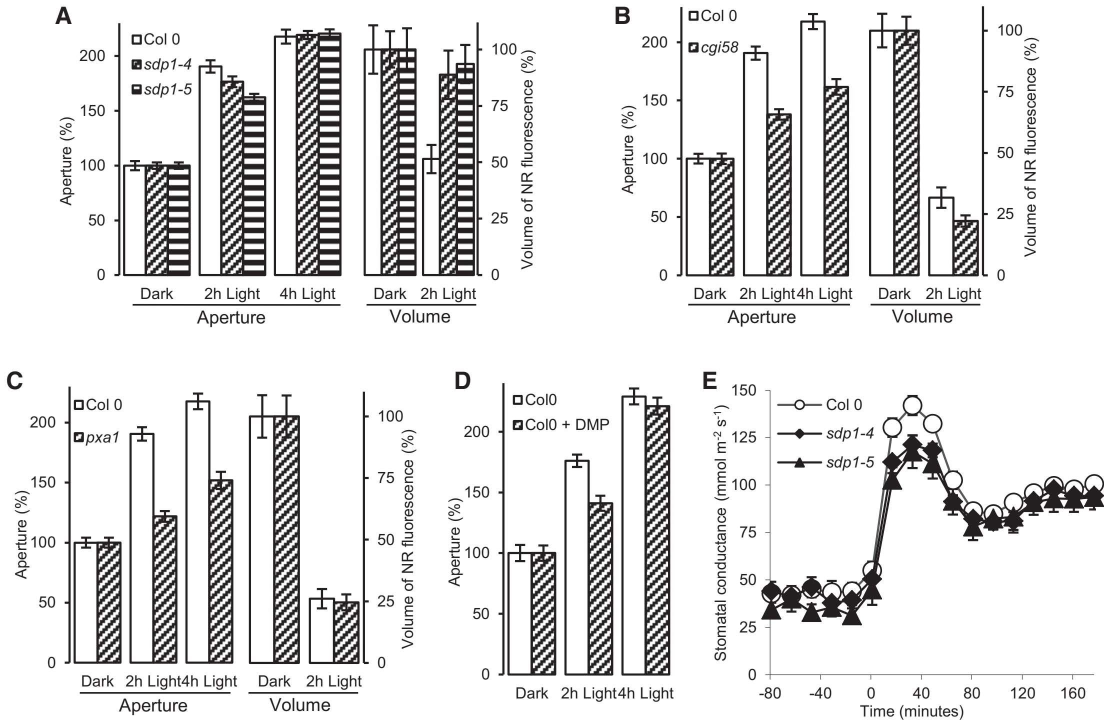

Figure 3. Mutants Carrying Lesions in TAG Catabolism Display Slower Light-Induced Stomatal Opening than Wild-Type

(A) *sdp1-4* and *1-5* have delayed stomatal opening ($p < 0.05$ at 2 hr; $p > 0.05$ at 4 hr; $n = 90$), and LD breakdown is greatly reduced ($p < 0.01$; $n = 54–62$).

(B and C) Light-induced stomatal opening is disrupted in *cgi58* (B; $p < 0.001$ at both time points; $n = 90$) and *pxa1* (C; $p < 0.001$ at both time points; $n = 90$). LD breakdown is slightly increased in *cgi58* ($p < 0.05$; $n = 57–85$), but not in *pxa1* ($p > 0.05$; $n = 70–97$).

(D) DMP delays light-induced stomatal opening ($p < 0.001$ at 2 hr; $p > 0.05$ at 4 hr; $n = 90$).

(E) *sdp1-4* and *1-5* have lower stomatal conductance immediately after a dark to light transition ($p < 0.05$; $n = 8$).

Error bars represent ±SE. See also Figures S2 and S4.

LD Imaging

Abaxial epidermal strips, detached 30 min pre-dawn, were incubated for 2 hr in dark then 2 hr in either light (100 μmol m^{-2} s^{-1} PFD) or dark. Strips were then incubated in 30 μM NR (Sigma) for 20 min and washed in KCl/MES buffer for 5 min before imaging. NR fluorescence was imaged with a Leica TCS SP5 confocal microscope equipped with argon laser, excitation 488 nm, emission 525–575 nm. Image stacks were taken every 0.5 μm and LD numbers and volume computed using Leica Advanced Fluorescence v3.1.0 (Leica), ImageJ 1.46r (NIH), and its plug-in 3D object counter [39]. For Figure 1A, imaging was as above except excitation was at 458 nm, LD emission was collected at 520–550 nm, and chlorophyll autofluorescence was collected at 700–800 nm. For Figure 1B, abaxial epidermal strips, detached 3 hr post-dawn, were incubated for 4 hr in either light (100 μmol m^{-2} s^{-1} PFD) or dark. NR fluorescence was imaged with a Zeiss Axiovert 200 M fluorescence microscope equipped with an Optigrid and Hamamatsu 1394 ORCA-ERA camera, excitation 450–490 nm, emission 500–550 nm, and volume was computed using Volocity v4.3.2 (Improvision).

Ratiometric pH Quantification in Intact Leaves under Blue Light Irradiation

Blue-light-induced changes in leaf pH_{apo} were measured using dark-adapted *Arabidopsis thaliana* plants that were subjected to continual blue light irradiation at 440 nm during quantification. For in vivo quantification of leaf pH_{apo}, 25 μM solution of the fluorescent pH indicator dye Oregon Green 488-dextran (Invitrogen) was infiltrated into the leaf apoplast of intact plants using a needleless syringe [25, 26]. All pH responses were monitored starting 2 hr after the dye infiltration to ensure evaporation of excess infiltrated water and normal gas exchange within the apoplast [25]. Fluorescence images were collected as a time series with a Leica inverted microscope (DMI6000B; Leica Microsystems) connected to a DFC camera

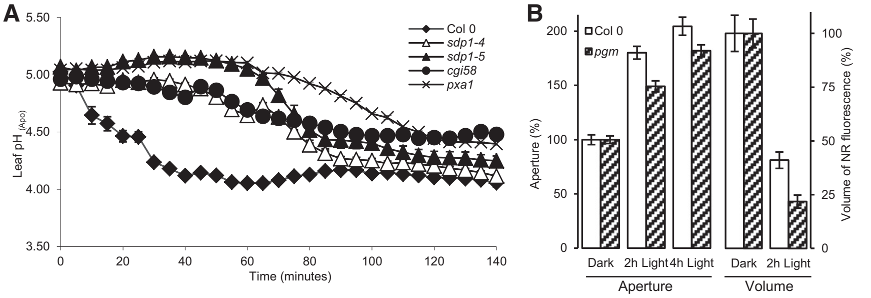

Figure 4. Blue-Light-Induced Apoplastic Acidification Is Delayed in TAG Metabolism Mutants, and Light-Induced Opening Is Impaired in the Starch-Deficient Mutant *pgm*

(A) Blue-light-induced leaf apoplastic acidification in *sdp1-4*, *sdp1-5*, *cgi-58*, *pxa1*, and WT ($n = 5$) and plotted over time.

(B) Light-induced stomatal opening and LD breakdown in the starch-deficient mutant *pgm1* ($n = 90$, $p < 0.05$ at 2 hr and $p < 0.01$ at 4 hr; $n = 89–93$, $p < 0.001$ for LD reduction). Error bars represent ±SE. See also Figure S3.

CellPress

(DFC 360FX; Leica Microsystems). An HXP lamp (HXP Short Arc Lamp; Osram) was used for illumination at excitation wavelengths 440/20 and 490/10 nm. The exposure time was 25 ms for both channels, and emission was collected at 535/25 nm. Blue light irradiation was stopped during image acquisition. The fluorescence ratio F_{490}/F_{440} was obtained as a measurement of pH on a pixel-by-pixel basis. Image analysis was carried out using LAS AF software (version 2.3.5; Leica Microsystems). Background values were subtracted at each channel. For conversion of the fluorescence ratio data into pH_{apo} values, an in vivo calibration was conducted as described elsewhere [27]. In brief, Oregon Green dye solutions were pH buffered with citric acid/sodium citrate ($3.0 \leq pH \leq 5.0$; 10 mM), MES ($5.5 \leq pH \leq 6.0$; 50 mM), and PIPES ($6.5 \leq pH \leq 7.5$; 50 mM) and infiltrated as above. The Boltzmann fit was chosen to fit sigmoidal curves to the calibration data. Atmospheric water vapor pressure deficit was 0.58 kPa during the entire experiment.

Gas Exchange Measurements

Stomatal conductance patterns of whole plants were recorded using a custom-built gas exchange device [40].

TAG Analysis

TAGs were extracted from ~30 mg fresh-frozen epidermal strips, detached either 1 hr pre-dawn or 2 or 3 hr post-dawn, and analyzed by LC/MS/MS [41]. On the basis of fluorescein diacetate fluorescence, we estimate that 98.9% of the guard cells were intact, non-ruptured, and viable, whereas the corresponding figure for epidermal pavement cells was 1.1%. A master peak identification list for TAGs, diacylglycerols, and galactolipids was generated from a combination of data-dependent MS/MS spectra and authentic standards using a software pipeline of the R (v2.11; https://www.r-project.org) packages XCMS [42], CAMERA [43], and custom scripts. This returned 14 TAG species, which were quantified as their ammoniated pseudomolecular ions relative to the ^{13}C-labeled triolein internal standard and expressed on a fresh weight basis. Throughout the text, TAGs are labeled as the non-regiospecific concatenation of three x:y fatty acyl moieties where x is number of carbons and y is number of double bonds.

Statistics

All data were analyzed using Student's t test or ANOVA with Tukey post hoc. Information on the in silico analysis is given in the Supplemental Experimental Procedures.

SUPPLEMENTAL INFORMATION

Supplemental Information includes Supplemental Experimental Procedures, four figures, and two tables and can be found with this article online at http://dx.doi.org/10.1016/j.cub.2016.01.019.

AUTHOR CONTRIBUTIONS

A.M.H. conceived and designed the experiments, interpreted the data, and wrote the paper; D.H.M. and J.L. designed and carried out experiments, interpreted the data, and wrote the paper; C.-M.G., A.N.D., T.L., H.H., H.K., and Z.H. carried out experiments and interpreted data; I.G. and M.V.M. designed experiments, interpreted data, and wrote the paper; and A.B. provided reagents, analyzed data, and contributed to writing the paper.

ACKNOWLEDGMENTS

A.M.H. acknowledges the BBSRC, the Royal Society, the Gatsby Charitable Foundation, and the Leverhulme Trust for the award of an Advanced Fellowship. Work in the lab of H.K. was supported by Estonian Research Council (IUT2-21) and by European Regional Fund (Centre of Excellence in Environmental Adaptation). Karl H. Mühling is acknowledged for providing access to the ratio imaging device, and we thank Christoph Plieth for advice on fitting the pH calibration data to a sigmoidal Boltzmann fit. The authors are grateful to Dr. Kent Chapman (University of North Texas) for the gift of the *cgi-58* seed.

Received: September 24, 2015
Revised: December 9, 2015
Accepted: January 7, 2016
Published: February 18, 2016

REFERENCES

1. Hetherington, A.M., and Woodward, F.I. (2003). The role of stomata in sensing and driving environmental change. Nature *424*, 901–908.
2. Yoo, C.Y., Pence, H.E., Hasegawa, P.M., and Mickelbart, M.V. (2009). Regulation of transpiration to improve crop water use. Crit. Rev. Plant Sci. *28*, 410–431.
3. Shimazaki, K., Doi, M., Assmann, S.M., and Kinoshita, T. (2007). Light regulation of stomatal movement. Annu. Rev. Plant Biol. *58*, 219–247.
4. Vavasseur, A., and Raghavendra, A.S. (2005). Guard cell metabolism and CO_2 sensing. New Phytol. *165*, 665–682.
5. Tominaga, M., Kinoshita, T., and Shimazaki, K. (2001). Guard-cell chloroplasts provide ATP required for $H^{(+)}$ pumping in the plasma membrane and stomatal opening. Plant Cell Physiol. *42*, 795–802.
6. Daloso, D.M., Antunes, W.C., Pinheiro, D.P., Waquim, J.P., Araújo, W.L., Loureiro, M.E., Fernie, A.R., and Williams, T.C. (2015). Tobacco guard cells fix CO_2 by both Rubisco and PEPcase while sucrose acts as a substrate during light-induced stomatal opening. Plant Cell Environ. *38*, 2353–2371.
7. Sato, N. (1985). Lipid biosynthesis in epidermal, guard and mesophyll cell protoplasts from leaves of *Vicia faba L*. Plant Cell Physiol. *26*, 805–811.
8. Sakaki, T., Satoh, A., Tanaka, K., Omasa, K., and Shimazaki, K.-I. (1995). Lipids and fatty acids in guard-cell protoplasts from *Vicia faba* leaves. Phytochemistry *40*, 1065–1070.
9. Sack, F.D. (1987). The development and structure of stomata. In Stomatal Function, E. Zeiger, G.D. Farquhar, and I.R. Cowan, eds. (Stanford University Press), pp. 59–90.
10. Greenspan, P., Mayer, E.P., and Fowler, S.D. (1985). Nile red: a selective fluorescent stain for intracellular lipid droplets. J. Cell Biol. *100*, 965–973.
11. Kinoshita, T., Doi, M., Suetsugu, N., Kagawa, T., Wada, M., and Shimazaki, K. (2001). Phot1 and phot2 mediate blue light regulation of stomatal opening. Nature *414*, 656–660.
12. Graham, I.A. (2008). Seed storage oil mobilization. Annu. Rev. Plant Biol. *59*, 115–142.
13. Kelly, A.A., van Erp, H., Quettier, A.L., Shaw, E., Menard, G., Kurup, S., and Eastmond, P.J. (2013). The sugar-dependent1 lipase limits triacylglycerol accumulation in vegetative tissues of Arabidopsis. Plant Physiol. *162*, 1282–1289.
14. Fan, J., Yan, C., Roston, R., Shanklin, J., and Xu, C. (2014). *Arabidopsis* lipins, PDAT1 acyltransferase, and SDP1 triacylglycerol lipase synergistically direct fatty acids toward β-oxidation, thereby maintaining membrane lipid homeostasis. Plant Cell *26*, 4119–4134.
15. Eastmond, P.J. (2006). SUGAR-DEPENDENT1 encodes a patatin domain triacylglycerol lipase that initiates storage oil breakdown in germinating Arabidopsis seeds. Plant Cell *18*, 665–675.
16. Footitt, S., Slocombe, S.P., Larner, V., Kurup, S., Wu, Y., Larson, T., Graham, I., Baker, A., and Holdsworth, M. (2002). Control of germination and lipid mobilization by COMATOSE, the Arabidopsis homologue of human ALDP. EMBO J. *21*, 2912–2922.
17. Hayashi, H., De Bellis, L., Hayashi, Y., Nito, K., Kato, A., Hayashi, M., Hara-Nishimura, I., and Nishimura, M. (2002). Molecular characterization of an Arabidopsis acyl-coenzyme a synthetase localized on glyoxysomal membranes. Plant Physiol. *130*, 2019–2026.
18. Zolman, B.K., Silva, I.D., and Bartel, B. (2001). The Arabidopsis pxa1 mutant is defective in an ATP-binding cassette transporter-like protein required for peroxisomal fatty acid beta-oxidation. Plant Physiol. *127*, 1266–1278.
19. Flügge, U.I., Häusler, R.E., Ludewig, F., and Gierth, M. (2011). The role of transporters in supplying energy to plant plastids. J. Exp. Bot. *62*, 2381–2392.

20. Jedd, G., and Chua, N.H. (2002). Visualization of peroxisomes in living plant cells reveals acto-myosin-dependent cytoplasmic streaming and peroxisome budding. Plant Cell Physiol. *43*, 384–392.

21. James, C.N., Horn, P.J., Case, C.R., Gidda, S.K., Zhang, D., Mullen, R.T., Dyer, J.M., Anderson, R.G.W., and Chapman, K.D. (2010). Disruption of the Arabidopsis CGI-58 homologue produces Chanarin-Dorfman-like lipid droplet accumulation in plants. Proc. Natl. Acad. Sci. USA *107*, 17833–17838.

22. Park, S., Gidda, S.K., James, C.N., Horn, P.J., Khuu, N., Seay, D.C., Keereetaweep, J., Chapman, K.D., Mullen, R.T., and Dyer, J.M. (2013). The α/β hydrolase CGI-58 and peroxisomal transport protein PXA1 coregulate lipid homeostasis and signaling in Arabidopsis. Plant Cell *25*, 1726–1739.

23. Brown, L.A., Larson, T.R., Graham, I.A., Hawes, C., Paudyal, R., Warriner, S.L., and Baker, A. (2013). An inhibitor of oil body mobilization in Arabidopsis. New Phytol. *200*, 641–649.

24. Roelfsema, M.R.G., Staal, M., and Prins, H.B.A. (1998). Blue light-induced apoplastic acidification of *Arabidopsis thaliana* guard cells: inhibition by ABA is mediated through protein phosphatases. Physiol. Plant. *103*, 466–474.

25. Geilfus, C.M., and Mühling, K.H. (2011). Real-time imaging of leaf apoplastic pH dynamics in response to NaCl stress. Front. Plant Sci. *2*, 13.

26. Geilfus, C.M., and Mühling, K.H. (2013). Ratiometric monitoring of transient apoplastic alkalinizations in the leaf apoplast of living *Vicia faba* plants: chloride primes and PM-H+-ATPase shapes NaCl-induced systemic alkalinizations. New Phytol. *197*, 1117–1129.

27. Geilfus, C.M., Mühling, K.H., Kaiser, H., and Plieth, C. (2014). Bacterially produced Pt-GFP as ratiometric dual-excitation sensor for in planta mapping of leaf apoplastic pH in intact *Avena sativa* and *Vicia faba*. Plant Methods *10*, 31.

28. Pracharoenwattana, I., Cornah, J.E., and Smith, S.M. (2005). Arabidopsis peroxisomal citrate synthase is required for fatty acid respiration and seed germination. Plant Cell *17*, 2037–2048.

29. Dave, A., and Graham, I.A. (2012). Oxylipin signaling: a distinct role for the jasmonic acid precursor cis-(+)-12-oxo-phytodienoic acid (cis-OPDA). Front. Plant Sci. *3*, 42.

30. Baker, A., Graham, I.A., Holdsworth, M., Smith, S.M., and Theodoulou, F.L. (2006). Chewing the fat: beta-oxidation in signalling and development. Trends Plant Sci. *11*, 124–132.

31. Theodoulou, F.L., Job, K., Slocombe, S.P., Footitt, S., Holdsworth, M., Baker, A., Larson, T.R., and Graham, I.A. (2005). Jasmonic acid levels are reduced in COMATOSE ATP-binding cassette transporter mutants. Implications for transport of jasmonate precursors into peroxisomes. Plant Physiol. *137*, 835–840.

32. Caspar, T., Huber, S.C., and Somerville, C. (1985). Alterations in growth, photosynthesis, and respiration in a starchless mutant of Arabidopsis thaliana (L) deficient in chloroplast phosphoglucomutase activity. Plant Physiol. *79*, 11–17.

33. Lascève, G., Leymarie, J., and Vavasseur, A. (1997). Alterations in light-induced stomatal opening in a starch-deficient mutant of *Arabidopsis thaliana* L. deficient in chloroplast phosphoglucomutase activity. Plant Cell Environ. *20*, 350–358.

34. Würtele, M., Jelich-Ottmann, C., Wittinghofer, A., and Oecking, C. (2003). Structural view of a fungal toxin acting on a 14-3-3 regulatory complex. EMBO J. *22*, 987–994.

35. Ruszala, E.M., Beerling, D.J., Franks, P.J., Chater, C., Casson, S.A., Gray, J.E., and Hetherington, A.M. (2011). Land plants acquired active stomatal control early in their evolutionary history. Curr. Biol. *21*, 1030–1035.

36. Doi, M., Kitagawa, Y., and Shimazaki, K. (2015). Stomatal blue light response is present in early vascular plants. Plant Physiol. *169*, 1205–1213.

37. Sack, F., and Paolillo, D.J. (1983). Structure and development of walls in Funaria stomata. Am. J. Bot. *70*, 1019–1030.

38. Shimada, T.L., and Hara-Nishimura, I. (2015). Leaf oil bodies are subcellular factories producing antifungal oxylipins. Curr. Opin. Plant Biol. *25*, 145–150.

39. Bolte, S., and Cordelières, F.P. (2006). A guided tour into subcellular co-localization analysis in light microscopy. J. Microsc. *224*, 213–232.

40. Kollist, T., Moldau, H., Rasulov, B., Oja, V., Rämma, H., Hüve, K., Jaspers, P., Kangasjärvi, J., and Kollist, H. (2007). A novel device detects a rapid ozone-induced transient stomatal closure in intact Arabidopsis and its absence in abi2 mutant. Physiol. Plant. *129*, 796–803.

41. Burgal, J., Shockey, J., Lu, C., Dyer, J., Larson, T., Graham, I., and Browse, J. (2008). Metabolic engineering of hydroxy fatty acid production in plants: RcDGAT2 drives dramatic increases in ricinoleate levels in seed oil. Plant Biotechnol. J. *6*, 819–831.

42. Smith, C.A., Want, E.J., O'Maille, G., Abagyan, R., and Siuzdak, G. (2006). XCMS: processing mass spectrometry data for metabolite profiling using nonlinear peak alignment, matching, and identification. Anal. Chem. *78*, 779–787.

43. Kuhl, C., Tautenhahn, R., Böttcher, C., Larson, T.R., and Neumann, S. (2012). CAMERA: an integrated strategy for compound spectra extraction and annotation of liquid chromatography/mass spectrometry data sets. Anal. Chem. *84*, 283–289.

Current Biology, Volume *26*

Supplemental Information

The Breakdown of Stored Triacylglycerols

Is Required during Light-Induced Stomatal Opening

Deirdre H. McLachlan, Jue Lan, Christoph-Martin Geilfus, Antony N. Dodd, Tony Larson, Alison Baker, Hanna Hõrak, Hannes Kollist, Zhesi He, Ian Graham, Michael V. Mickelbart, and Alistair M. Hetherington

Supplemental Data

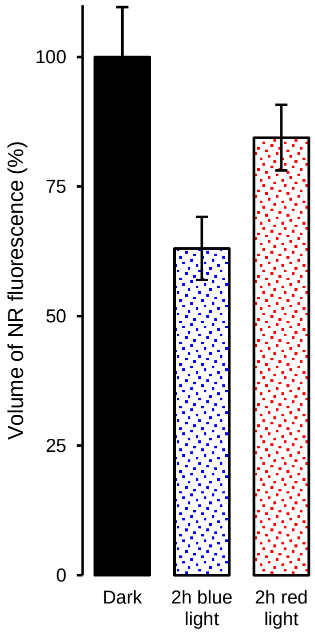

Figure S1. Related to Figure 1. Wavelength specificity of LD reduction. 10 µmol m^{-2} s^{-1} of blue light (λ=470nm) causes significant breakdown of LD ($p<0.01$), while the same amount of red light (λ=660nm) does not ($p>0.05$). Error bars = ± S.E., n=90.

A

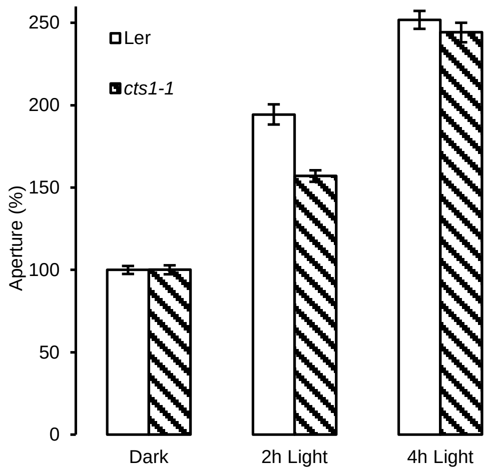

B

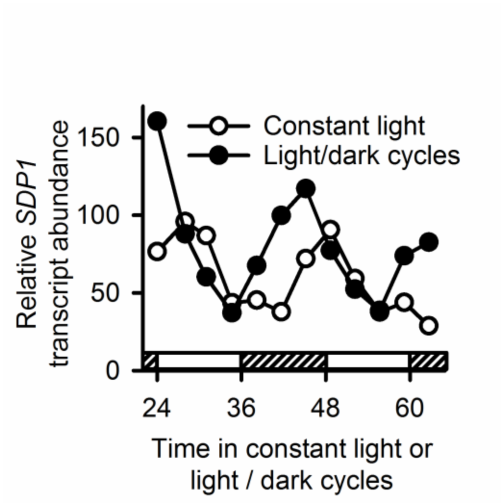

C

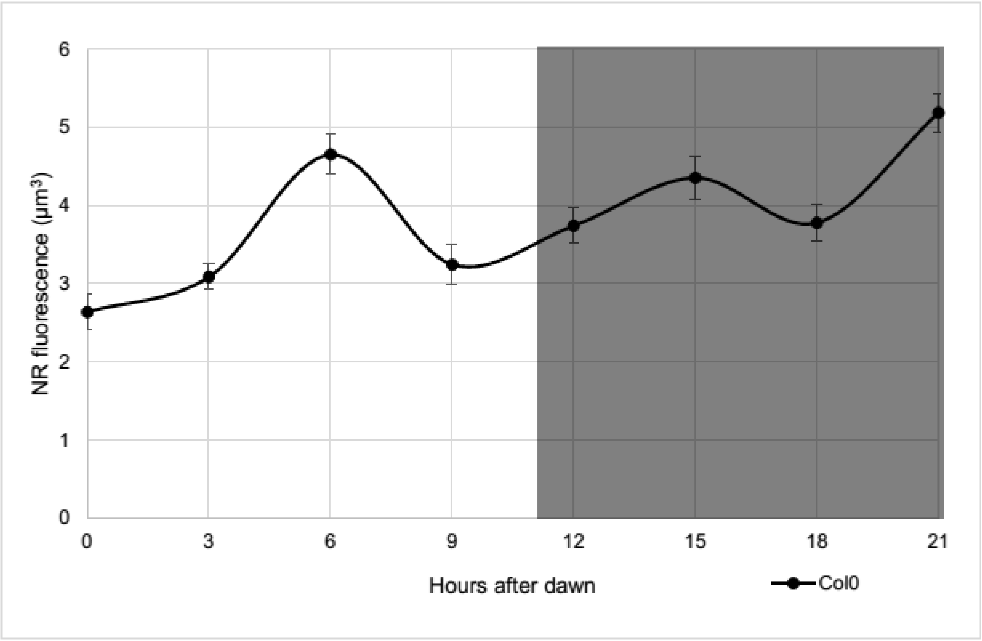

Figure S2. Related to Figure 3. A - The *cts1-1* mutant displays slower light-induced stomatal opening than WT. Error bars = ± SE, n=90, $p<0.001$ at 2h, $p>0.05$ at 4h. B - *SDP1* transcript abundance oscillates under light-dark cycles and constant light, peaking around actual or subjective dawn. Data were obtained by interrogation of microarray data using Diurnal [S1]. RNA timecourses were obtained from 35-day old compost grown seedlings [S2] and 8-day old agar grown seedlings [S3] for light/dark and constant light experiments, respectively. C - Change of LD volume in guard cells through a 24 h diurnal cycle. Error bars = ± SE, n=121-142 per timepoint

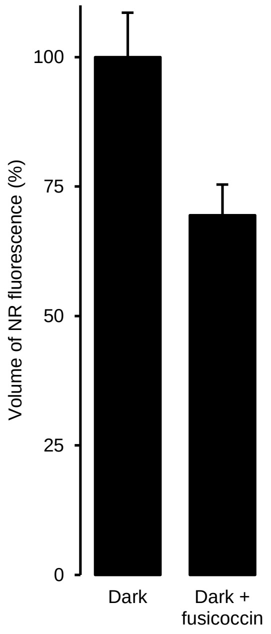

Figure S3. Related to Figure 4. Effect of fusicoccin on LD. Dark-incubated guard cells showed significant LD breakdown after 20μM fusicoccin treatment for 3 h. $p<0.01$, $n=90$, error bars = ± SE.

A

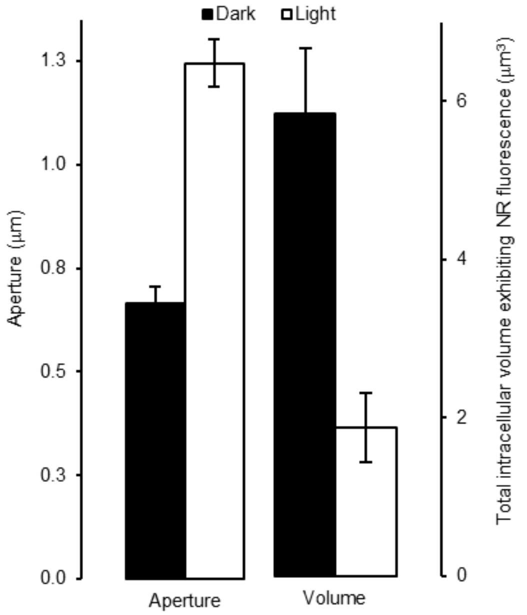

B

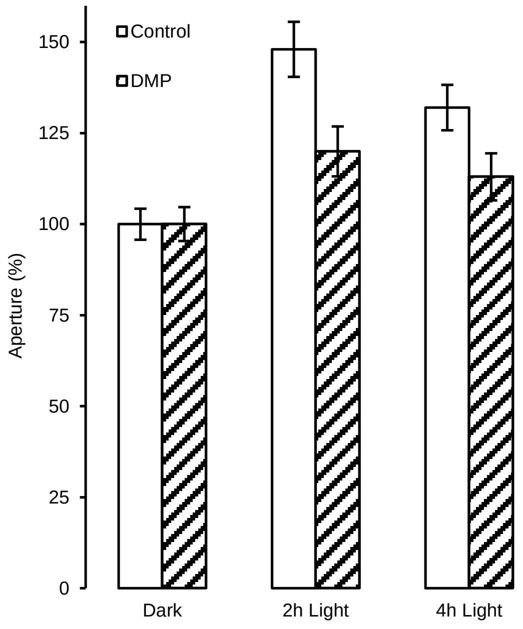

Figure S4. Related to Figure 3. Light-induced stomatal opening in *Selaginella* is associated with a reduction in abundance of LDs and is inhibited by the LD breakdown inhibitor DMP. (A) Light induced stomatal opening is associated with a decrease in NR fluorescence (n=90, p<0.001, error bars = ±SE). (B) Light-induced stomatal opening is inhibited by DMP (n=90, p<0.001, error bars = ± SE)

Table S1. Abundance of total TAG and individual TAG molecular species pre and post dawn (for full details see methods).

TAG species	Time	ng/mg FW	S.E.	Change (% of predawn)	p value	No. of samples
18:2-18:3-18:3	predawn	3.7	0.65	48	0.011	13
	postdawn	1.8	0.32			14
18:2-18:2-18:3	predawn	2.5	0.41	50	0.013	13
	postdawn	1.3	0.22			14
18:3-18:3-18:3	predawn	1.6	0.31	54	0.048	13
	postdawn	0.9	0.17			14
16:0-18:2-18:2	predawn	1.4	0.08	66	0.032	2
	postdawn	0.9	0.04			2
16:0-18:3-18:3	predawn	0.8	0.19	69	0.267	13
	postdawn	0.6	0.13			14
18:2-18:2-18:2	predawn	1.4	0.17	82	0.314	6
	postdawn	1.2	0.17			8
18:1-18:1-18:2	predawn	2.0	0.94	87	0.786	13
	postdawn	1.7	0.26			14
16:0-16:0-18:2	predawn	0.5	0.16	106	0.883	6
	postdawn	0.5	0.13			8
18:0-18:0-18:2	predawn	0.9	0.13	114	0.498	6
	postdawn	1.1	0.13			8
18:0-18:2-18:3	predawn	0.2	0.05	114	0.645	6
	postdawn	0.3	0.05			8
18:1-18:2-18:2	predawn	1.9	0.42	115	0.644	6
	postdawn	2.1	0.40			8
16:0-18:1-18:2	predawn	1.5	0.43	118	0.644	6
	postdawn	1.8	0.37			8
18:0-18:2-18:2	predawn	0.5	0.15	125	0.531	6
	postdawn	0.6	0.13			8
16:0-18:1-18:1	predawn	2.0	0.65	130	0.478	6
	postdawn	2.6	0.53			8
**Total**	**predawn**	**14.5**	**2.84**	**76**	**0.355**	**13**
	**postdawn**	**11.1**	**2.18**			**14**

Table S2.

Expression levels of TAG lipolysis, peroxisomal fatty acid beta-oxidation, glyoxylate cycle and gluconeogenesis related genes in Col-0 derived guard cell and leaf samples. The genes are grouped according to function and the order proceeds from lipolysis through the various steps and pathways to gluconeogenesis. Raw microarray data was downloaded and independently processed. Fold change was calculated to represent the comparison of the 2 sample types (guard cell and leaf) that had been maintained either in the presence or absence of exogenous ABA. Gene descriptions are from the latest version of the Arabidopsis genome annotation TAIR10 (http://www.arabidopsis.org/portals/genAnnotation/gene_structural_annotation/annotation_data.jsp).

Supplemental Experimental Procedures

Microarray data in Table S2. Unprocessed Affymetrix microarray data from Col-0 guard cell and leaf samples [S4] was downloaded from the gene expression omnibus (GEO) at NCBI using accession No. GSE19520 (http://www.ncbi.nlm.nih.gov/geo/query/acc.cgi?acc=GSE19520). The data were processed and normalised using the MAS5 algorithm in R/bioconductor with a mean target intensity of 500. A comprehensive list of genes involved in TAG lipolysis, peroxisomal fatty acid beta-oxidation, the glyoxylate cycle and gluconeogenesis was assembled from the Arabidopsis Acyl-Lipid Metabolism Website (ARALIP, http://aralip.plantbiology.msu.edu/pathways/triacylglycerol_fatty_acid_degradation,) [S5] and the compiled list of genes involved in storage oil mobilisation [S6]. The CGI-58 gene with a proposed role in neutral lipid homeostasis is also included [S7]. The expression values of these genes were calculated by averaging the triplicate measurements and a fold change was used to represent the comparison of the 2 sample types (guard cell and leaf) that had been maintained either in the presence or absence of exogenous ABA.

Supplemental References

S1. Mockler, T.C., Michael, T.P., Priest, H.D., Shen, R., Sullivan, C.M., Givan, S.A., McEntree, C., Kay, S.A. and Chory, J. (2007). The DIURNAL project: diurnal and circadian expression profiling, model-based pattern matching and promoter analysis. *Cold Spring Harb. Symp. Quant. Biol. 72*, 353-363.

S2. Edwards, K.D., Anderson, P.E., Hall, A., Salathia, N.S., Locke, J.C.W., Lynn, J.R., Straume, M., Smith, J.Q., and Millar, A.J. (2006). FLOWERING LOCUS C mediates natural variation in the high-temperature response of the Arabidopsis circadian clock. Plant Cell *18*, 639-650.

S3. Blasing, O.E., Gibon, Y., Gunther, M., Hohne, M., Morcuende, R., Osuna, D., Thimm, O., Usadel, B., Scheible, W.R., and Stitt, M. (2005). Sugars and circadian regulation make major contributions to the global regulation of diurnal gene expression in Arabidopsis. Plant Cell *17*, 3257-3281.

S4. Wang, R.S., Pandey, S., Li, S., Gookin, T.E., Zhao, Z.X., Albert, R., and Assmann, S.M. (2011). Common and unique elements of the ABA-regulated transcriptome of Arabidopsis guard cells. BMC Genomics *12*:216, doi: 10.1186/1471-2164-12-216.

S5. Li-Beisson Y., Shorrosh B., Beisson F., Andersson M.X., Arondel V., Bates P.D., Baud S., Bird D., DeBono A., Durrett T.P., Franke R.B., Graham I.A., Katayama K., Kelly A.A., Larson T., Markham J.E., Miquel M., Molina I., Nishida I., Rowland O., Samuels L., Schmid K.M., Wada H., Welti R., Xu C. Zallot R., and Ohlrogge J. (2010) Acyl-Lipid Metabolism. *The Arabidopsis Book* 8:e0133. doi:10.1199/tab.0133

S6. Graham, I.A. (2008). Seed storage oil mobilization. Annu Rev Plant Biol *59*, 115-142.

S7. James, C.N., Horn, P.J., Case, C.R., Gidda, S.K., Zhang, D.Y., Mullen, R.T., Dyer, J.M., Anderson, R.G.W., and Chapman, K.D. (2010). Disruption of the Arabidopsis CGI-58 homologue produces Chanarin-Dorfman-like lipid droplet accumulation in plants. P Natl Acad Sci USA *107*, 17833-17838.
